# Drought-responsive WRKY transcription factor genes *TaWRKY1* and *TaWRKY33* from wheat confer drought and/or heat resistance in *Arabidopsis*

**DOI:** 10.1186/s12870-016-0806-4

**Published:** 2016-05-23

**Authors:** Guan-Hua He, Ji-Yuan Xu, Yan-Xia Wang, Jia-Ming Liu, Pan-Song Li, Ming Chen, You-Zhi Ma, Zhao-Shi Xu

**Affiliations:** Institute of Crop Science, Chinese Academy of Agricultural Sciences (CAAS)/National Key Facility for Crop Gene Resources and Genetic Improvement, Key Laboratory of Biology and Genetic Improvement of Triticeae Crops, Ministry of Agriculture, Beijing, 100081 China; Shijiazhuang Academy of Agricultural and Forestry Sciences, Research Center of Wheat Engineering Technology of Hebei, Shijiazhuang, Hebei 050041 China

**Keywords:** Drought tolerance, WRKY transcription factor, Stress response mechanisms, Thermotolerance, *Triticum aestivum*

## Abstract

**Background:**

Drought stress is one of the major causes of crop loss. WRKY transcription factors, as one of the largest transcription factor families, play important roles in regulation of many plant processes, including drought stress response. However, far less information is available on drought-responsive WRKY genes in wheat (*Triticum aestivum* L.), one of the three staple food crops.

**Results:**

Forty eight putative drought-induced WRKY genes were identified from a comparison between *de novo* transcriptome sequencing data of wheat without or with drought treatment. *TaWRKY1* and *TaWRKY33* from WRKY Groups III and II, respectively, were selected for further investigation. Subcellular localization assays revealed that TaWRKY1 and TaWRKY33 were localized in the nuclei in wheat mesophyll protoplasts. Various abiotic stress-related *cis*-acting elements were observed in the promoters of *TaWRKY1* and *TaWRKY33*. Quantitative real-time PCR (qRT-PCR) analysis showed that *TaWRKY1* was slightly up-regulated by high-temperature and abscisic acid (ABA), and down-regulated by low-temperature. *TaWRKY33* was involved in high responses to high-temperature, low-temperature, ABA and jasmonic acid methylester (MeJA). Overexpression of *TaWRKY1* and *TaWRKY33* activated several stress-related downstream genes, increased germination rates, and promoted root growth in *Arabidopsis* under various stresses. *TaWRKY33* transgenic *Arabidopsis* lines showed lower rates of water loss than *TaWRKY1* transgenic *Arabidopsis* lines and wild type plants during dehydration. Most importantly, *TaWRKY33* transgenic lines exhibited enhanced tolerance to heat stress.

**Conclusions:**

The functional roles highlight the importance of WRKYs in stress response.

**Electronic supplementary material:**

The online version of this article (doi:10.1186/s12870-016-0806-4) contains supplementary material, which is available to authorized users.

## Background

Being unable to move, plants have developed a series of complex mechanisms to cope with abiotic and biotic stresses. Recognition of stress cues and transduction of signals to activate adaptive responses and regulation of stress-related genes are key steps leading to plant stress tolerance [1–4].

Due to the potential impact on agricultural production much attention has been focused on abiotic stress factors. Abiotic stresses initiate the synthesis of different types of proteins, including transcription factors, enzymes, molecular chaperones, ion channels, and transporters [5]. Transcriptional regulation mechanisms play a critical role in plant development and responses to environmental stimuli [4, 6, 7]. Transcription factors, with specific DNA-binding domains (DBD) and *trans*-acting functional domains, can combine with specific DNA sequences to activate or inhibit transcription of downstream genes. Using transcription factors to improve the tolerance of plants to abiotic stresses is a promising strategy due to the ability of transcription factors to modulate a set of genes through binding to either promoter or enhancer region of a gene [8]. Overexpression of constitutive active *DREB2A* which had a transcriptional activation domain between residues 254 and 335 resulted in significant drought stress tolerance through regulates expression of many water stress-inducible genes [9]. In our previous study, *GmHsf-34* gene improved drought and heat stresses tolerance in *Arabidopsis* plants [10]. These studies indicate the potential for improvement of abiotic stress tolerance in plants through transcriptional regulation.

WRKY transcription factors, one of the ten largest transcription factor families, are characterized by a highly conserved WRKYGQK heptapeptide at the N-terminus and a zinc finger-like motif at the C-terminus [11]. Conservation of the WRKY domain is mirrored by a remarkable conservation of its cognate binding site, the W box (TTGACC⁄T) [11–13]. A few WRKY proteins which show slight variations in the heptapeptide WRKYGQK motif can not bind the W box and may bind the WK box (TTTTCCAC) [14–17]. WRKYs are divided into three groups based on the number of WRKY domains and type of zinc finger motif. The first group has two WRKY domains. Groups II and III have a single WRKY domain and are distinguished according to the type of zinc finger motif [17]. Groups I and II share the same C_2_H_2_ zinc finger motif whereas group III contains a C_2_-HC-type motif [18]. Later, according to a more accurate phylogenetic analysis, Zhang and Wang divided WRKY factors into Groups I, IIa + IIb, IIc, IId + IIe, and III with Group II genes not being monophyletic [12].

Increasing data indicates that WRKY genes are rapidly induced by pathogen infection and exogenous phytohormones [19–25]. Forty nine of 72 *Arabidopsis* WRKY genes were differentially regulated after infection by *Pseudomonas syringae* or SA treatment [26]. Transcript abundance of 13 canola WRKY genes changed after pathogen infection [15]. Similarly, 28 grape WRKY genes showed various transcription expression in response to biotic stress caused by grape white rot and/or salicylic acid (SA). Among them 16 WRKY genes were upregulated by both pathogenic white rot bacteria and SA, indicating that these WRKY genes participated in the SA-dependent defense signal pathway [27]. Heterologous expression of *OsWRKY6* activated defense-related genes and enhanced resistance to pathogens in *Arabidopsis* [28]. Recently, it was reported that the OsMKK4-OsMPK3/OsMPK6 cascade regulates transactivation activity of OsWRKY53, and a phospho-mimic mutant of *OsWRKY53* resulted in further-enhanced disease resistance against the blast fungus in rice compared to native *OsWRKY53* [24].

In comparison with research progress on biotic stresses, the functions of WRKYs in abiotic stresses are far less known [29–36]. Increasing numbers of reports are showing that WRKYs respond to abiotic stress and abscisic acid (ABA) signaling in plants [37–41]. Several *Arabidopsis* WRKY genes can be induced by drought and/or cold stress [42, 43]. *AtWRKY46* regulated osmotic stress responses and stomatal movement independently in *Arabidopsis* [44]. *OsWRKY08* improved the osmotic stress tolerance of transgenic *Arabidopsis* through positive regulation of the expression of ABA-independent abiotic stress responsive genes [45]. Overexpression and RNAi analysis demonstrated that *GmWRKY27* improved salt and drought tolerance in transgenic soybean hairy roots by inhibits expression of a downstream gene *GmNAC29* which was a negative factor of stress tolerance [46]. Therefore, WRKYs play a broad-spectrum regulatory role as positive and negative regulators in response to biotic and abiotic stresses, senescence, seed development and seed germination [17, 25, 47].

Drought stress is one of the most severe environmental factors restricting crop distribution and production. The molecular mechanisms underlying plant tolerance to drought stress are still not fully understood because of the complex nature [48]. Bread wheat (*Triticum aestivum* L.) is one of the most widely cultivated and important food crops in the world. Drought affects growth and productivity of wheat, and reduces yields worldwide. It was recently reported that wheat *TaWRKY2* and *TaWRKY19* conferred tolerance to drought stress in transgenic plants [49]. To investigate putative drought-mediated WRKY genes, we performed *de novo* transcriptome sequencing of drought-treated wheat, and identified 48 wheat drought-responsive WRKY genes. We further investigated stress tolerance conferred by *TaWRKY1* and *TaWRKY33* in transgenic *Arabidopsis*. The present study investigated the possibility of improving stress tolerance in plants by screening stress responsive candidate genes.

## Results

### Identification of drought-responsive WRKY genes in wheat

In order to identify WRKY genes regulated by drought, we compared wheat *de novo* transcriptome sequencing data with or without drought treatment. A pairwise comparison of drought vs. without drought treatments revealed 48 WRKYs showing significant up- or down-regulation in transcription level (more than a twofold change) (Table 1). Nucleic acid sequences of 48 WRKYs in wheat were listed in Additional file 1: Table S1.

**Table 1:** Drought-induced responsive WRKY genes in wheat

Gene name	Gene ID	CK	Drought	Log fold change	Up/Down	FDR
*TaWRKY1*	Unigene50292_All	1	16	4.17	Up	1.51E-04
*TaWRKY2*	CL2151.Contig2_All	1	18	4.00	Up	4.90E-04
*TaWRKY3*	CL7466.Contig1_All	65	818	3.65	Up	1.51E-165
*TaWRKY4*	Unigene9495_All	7	80	3.51	Up	4.36E-16
*TaWRKY5*	Unigene24182_All	43	485	3.50	Up	1.16E-94
*TaWRKY6*	CL15640.Contig3_All	66	609	3.21	Up	7.22E-110
*TaWRKY7*	Unigene23958_All	35	311	3.15	Up	2.17E-55
*TaWRKY8*	CL7466.Contig2_All	108	928	3.10	Up	3.97E-162
*TaWRKY9*	CL2311.Contig1_All	46	350	2.93	Up	6.18E-58
*TaWRKY10*	CL2960.Contig4_All	11	78	2.83	Up	6.39E-13
*TaWRKY11*	CL9014.Contig1_All	375	2331	2.64	Up	0
*TaWRKY12*	CL2311.Contig2_All	17	98	2.53	Up	2.58E-14
*TaWRKY13*	CL15640.Contig5_All	125	643	2.36	Up	1.07E-83
*TaWRKY14*	CL2151.Contig1_All	35	179	2.35	Up	1.25E-23
*TaWRKY15*	CL15640.Contig2_All	136	675	2.31	Up	1.51E-85
*TaWRKY16*	CL321.Contig3_All	58	276	2.25	Up	3.32E-34
*TaWRKY17*	Unigene47896_All	14	64	2.19	Up	3.05E-08
*TaWRKY18*	CL2151.Contig3_All	11	49	2.16	Up	2.22E-06
*TaWRKY19*	CL4329.Contig1_All	695	2919	2.07	Up	0
*TaWRKY20*	CL3634.Contig1_All	26	106	2.03	Up	5.50E-12
*TaWRKY21*	CL14217.Contig1_All	731	2775	1.92	Up	1.73E-277
*TaWRKY22*	Unigene45898_All	13	48	1.88	Up	2.04E-05
*TaWRKY23*	CL9014.Contig2_All	294	1079	1.88	Up	1.71E-104
*TaWRKY24*	Unigene23130_All	874	2872	1.72	Up	1.76E-245
*TaWRKY25*	CL9014.Contig3_All	70	230	1.72	Up	3.05E-20
*TaWRKY26*	CL213.Contig2_All	43	126	1.55	Up	5.17E-10
*TaWRKY27*	CL9014.Contig6_All	41	118	1.53	Up	3.20E-09
*TaWRKY28*	Unigene27690_All	86	242	1.49	Up	1.56E-17
*TaWRKY29*	CL14321.Contig2_All	210	549	1.39	Up	7.32E-35
*TaWRKY30*	CL15640.Contig4_All	332	844	1.35	Up	6.48E-51
*TaWRKY31*	CL15640.Contig8_All	50	127	1.34	Up	2.53E-08
*TaWRKY32*	CL213.Contig3_All	61	153	1.33	Up	1.14E-09
*TaWRKY33*	Unigene22134_All	2632	6548	1.31	Up	0
*TaWRKY34*	CL1516.Contig3_All	336	833	1.31	Up	3.13E-48
*TaWRKY35*	CL15191.Contig2_All	211	519	1.30	Up	6.40E-30
*TaWRKY36*	CL9113.Contig1_All	87	196	1.17	Up	3.61E-10
*TaWRKY37*	CL9910.Contig2_All	98	214	1.13	Up	2.07E-10
*TaWRKY38*	Unigene13575_All	126	275	1.13	Up	3.97E-13
*TaWRKY39*	CL16569.Contig1_All	125	268	1.10	Up	2.23E-12
*TaWRKY40*	CL9014.Contig5_All	498	1013	1.02	Up	8.10E-40
*TaWRKY41*	CL1681.Contig3_All	108	51	-1.07	Down	2.59E-05
*TaWRKY42*	CL14934.Contig1_All	1552	412	-1.90	Down	1.85E-152
*TaWRKY43*	CL14934.Contig2_All	700	184	-1.92	Down	1.02E-69
*TaWRKY44*	CL8633.Contig1_All	172	39	-2.13	Down	4.13E-20
*TaWRKY45*	Unigene25087_All	66	8	-3.03	Down	9.61E-12
*TaWRKY46*	Unigene39119_All	26	2	-3.69	Down	8.13E-06
*TaWRKY47*	Unigene32932_All	16	1	-3.99	Down	5.15E-04
*TaWRKY48*	Unigene33182_All	20	1	-4.31	Down	4.81E-05

CK, mean of sample without drought treatment

Log fold change, log2 (Drought/CK)

*FDR* false discovery rate

**Fig. 1:**
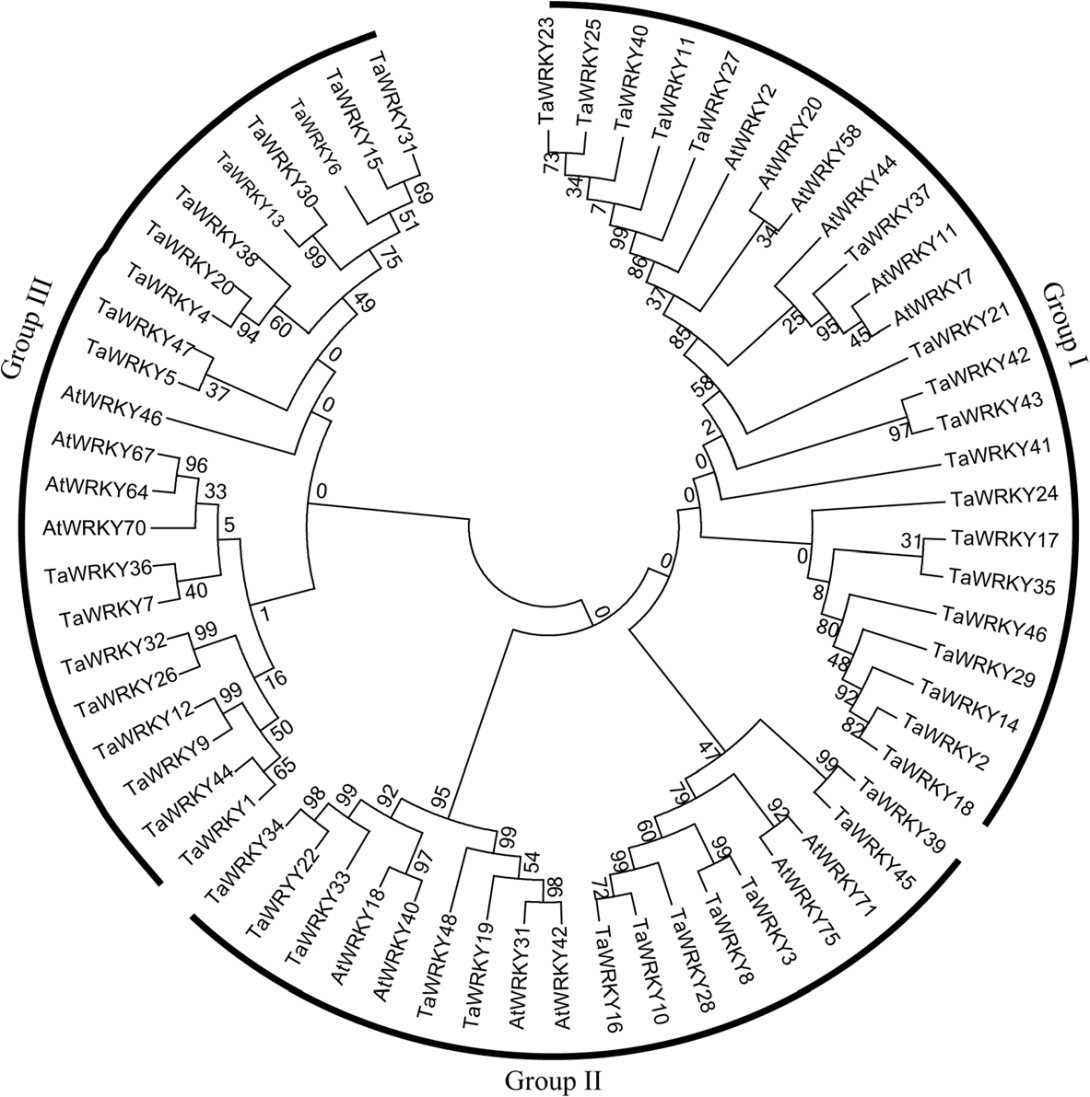
Maximum likelihood phylogenetic tree of drought-responsive WRKY genes in wheat and 16 AtWRKY proteins. The phylogenetic tree was based on comparisons of amino acid sequences and produced by MEGA 5.1 software

To investigate the evolutionary relationships of the drought-induced wheat WRKYs with previously reported WRKYs, a phylogenic tree was constructed using MEGA5.1. Twenty four drought-induced wheat WRKYs belonged to Group II, 15 to Group III, and nine to Group I (Fig. 1).

### Sequence analysis of *TaWRKY1* and *TaWRKY33*

**Fig. 2:**
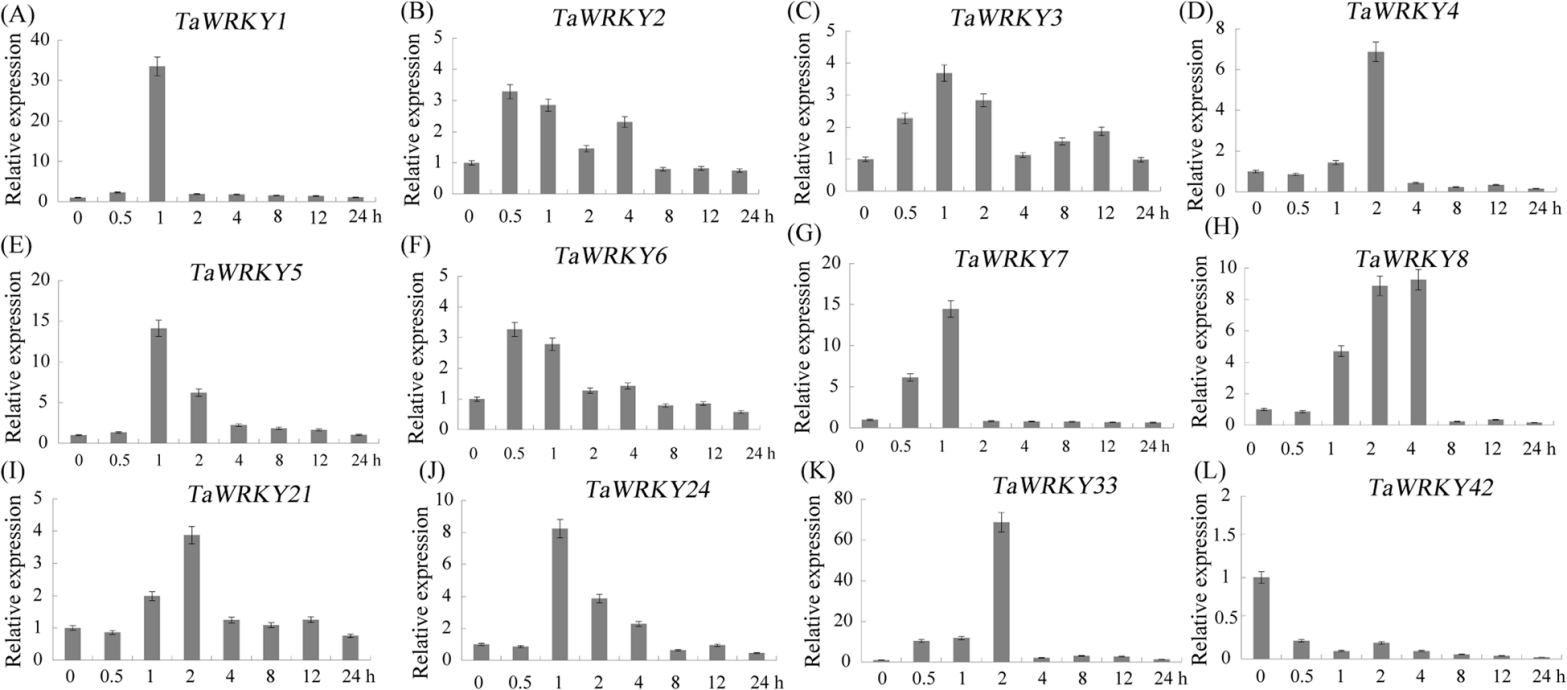
Expression patterns of 12 wheat WRKY genes under drought stress. These 12 wheat WRKY genes include *TaWRKY1* (**a**), *TaWRKY2* (**b**), *TaWRKY3* (**c**), *TaWRKY4* (**d**), *TaWRKY5* (**e**), *TaWRKY6* (**f**), *TaWRKY7* (**g**), *TaWRKY8* (**h**), *TaWRKY21* (**i**), *TaWRKY24* (**j**), *TaWRKY33* (**k**) and *TaWRKY42* (**l**). The ordinates are fold changes, and the horizontal ordinate is treatment time. The *actin* gene was used as an internal reference. The data are representative of three independent experiments

Among the 48 drought-induced wheat WRKY genes, *TaWRKY1* to *TaWRKY8* showed the largest transcript differences, being up-regulated more than three-log fold (log2 (Drought/CK)) and *TaWRKY21/24/33/42* showed the largest background transcript levels among all WRKY genes regulated by drought (Table 1). The drought stress expression patterns of these 12 wheat WRKY genes were further investigated. As shown in Fig. 2, *TaWRKY1* and *TaWRKY33* gave high responses to drought stress, peaking at more than 30-fold at one and two h, respectively. These genes were selected for further investigation.

**Fig. 3:**
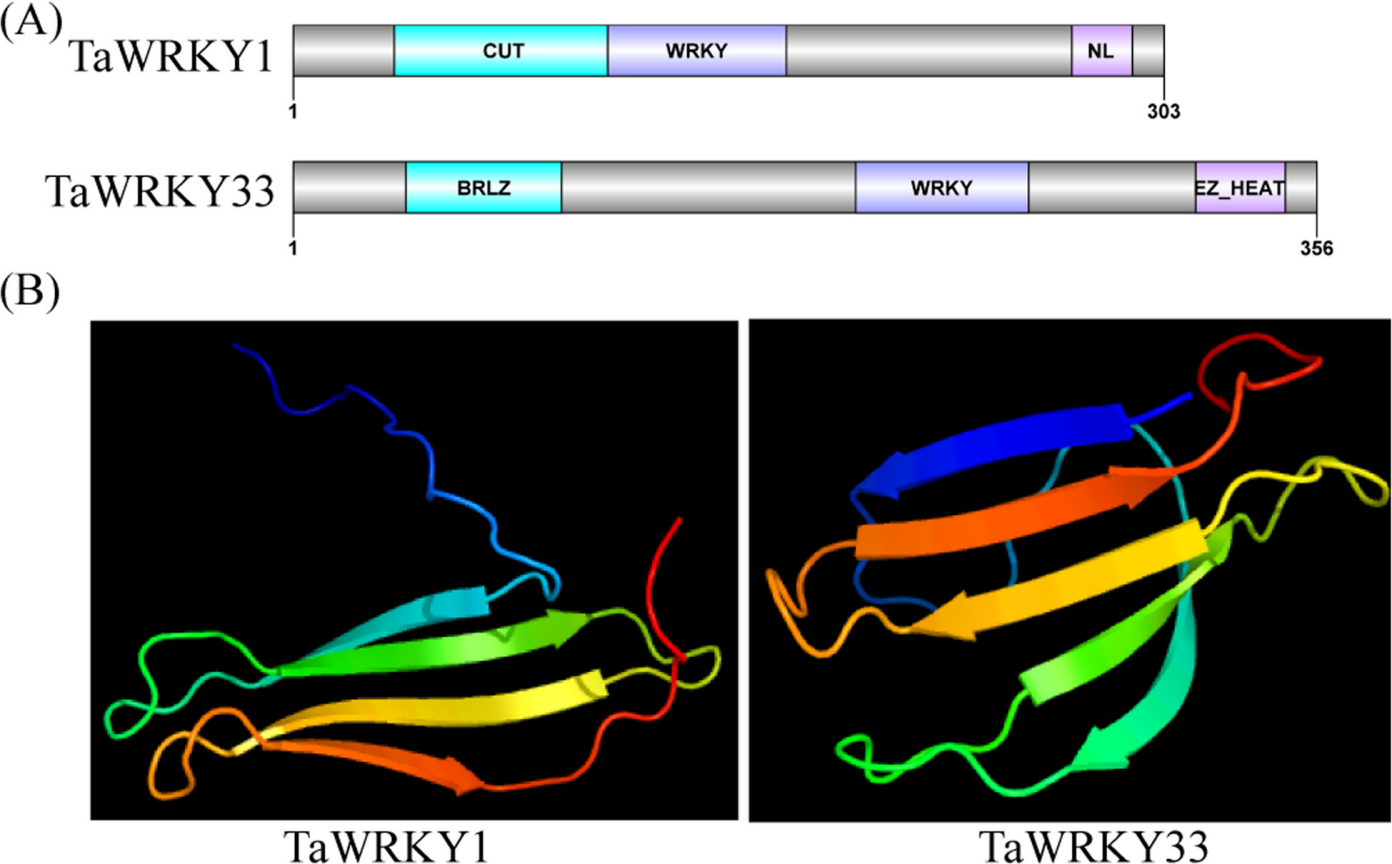
Domain organization (**a**) and tertiary structures (**b**) of TaWRKY1 and TaWRKY33

*TaWRKY1* contained a 912 bp open reading frame (ORF) encoding a 303 amino acid protein of 32.41 kDa with p*I* 4.68. The ORF of *TaWRKY33* was 1071 bp encoding a 38.8 kDa protein with p*I* 8.17. The predicted amino acid sequences of TaWRKY1 and TaWRKY33 possessed one WRKY domain with the highly conserved WRKYGQK motif, but two different deduced zinc finger motifs (C–X_7_–C–X_23_–H–X_1_–C and C–X_5_–C–X_23_–H–X_1_–H), respectively. TaWRKY1 contained an N-terminal CUT domain (amino acids 36 to 112) and a C-terminal NL domain (amino acids 271 to 292) according to SMART (Fig. 3a). TaWRKY33 contained an N-terminal basic region leucin zipper (BRLZ) domain (amino acids 40 to 94) and a C-terminal E-Z type HEAT Repeat (EZ_HEAT) domain (amino acids 314 to 345) (Fig. 3a). A four-stranded β-sheet with a zinc-binding pocket formed by conserved Cys/His residues was present in WRKY domains in the tertiary structures of TaWRKY1 and TaWRKY33 (Fig. 3b). We searched for WRKY homologies in NCBI using TaWRKY1 as a query. Amino acid sequence alignment showed that TaWRKY1 shared the highest identity (100 %) with AetWRKY70 (Aet07853) from the wild diploid *Aegilops tauschii* (2n = 14; DD), a progenitor of hexaploid wheat (*T. aestivum*; 2n = 6 × = 42; AABBDD) [50], suggesting that *TaWRKY1* was located in a D-genome chromosome. No candidate with complete identity to *TaWRKY33* was found in the genomic databases of *A. tauschii* and *Triticum urartu* (2n = 14; AA), the A-genome Progenitor. Therefore, *TaWRKY33* might be located in a B chromosome.

### TaWRKY1 and TaWRKY33 were localized in the nucleus

**Fig. 4:**
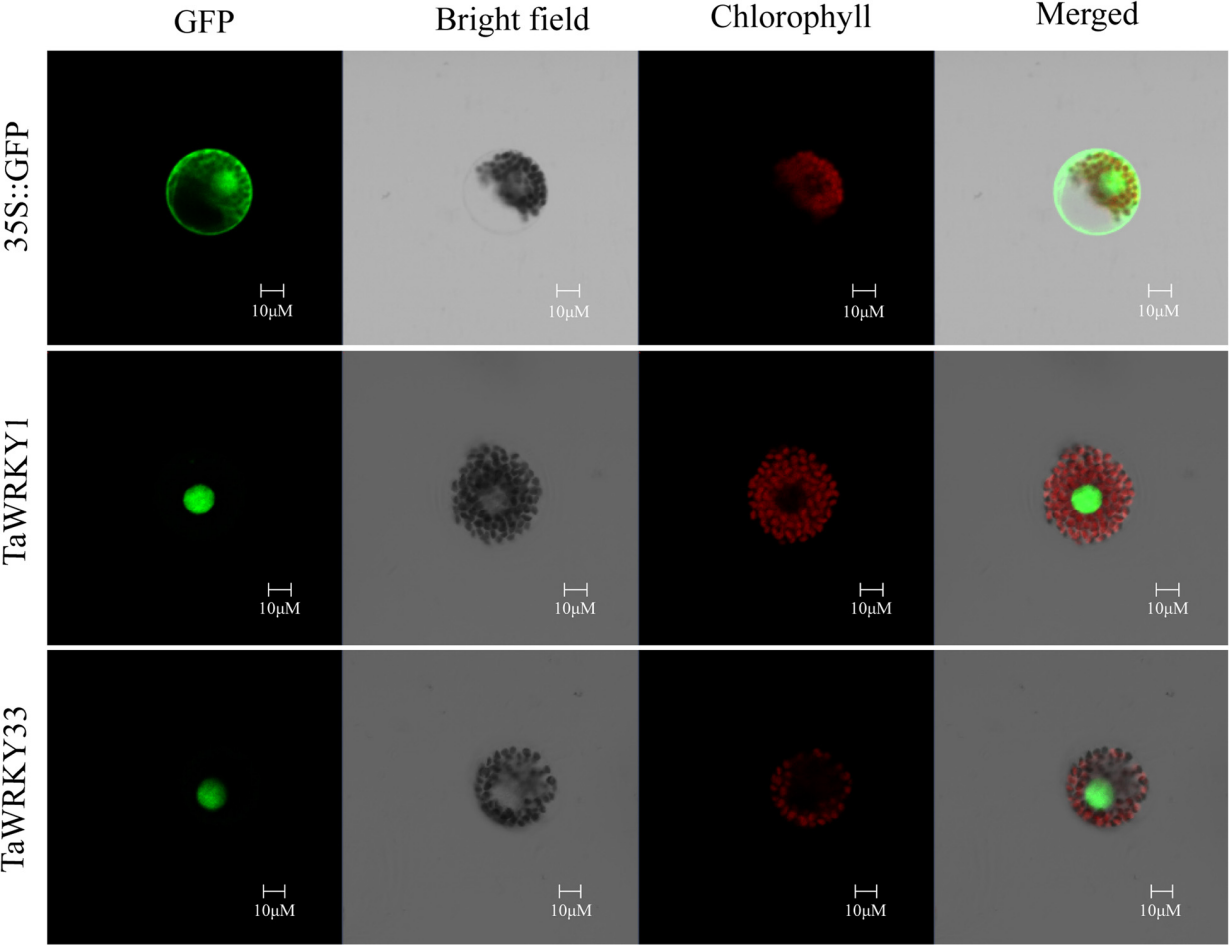
Subcellular localization of the TaWRKY1 and TaWRKY33 proteins. 35S::TaWRKY1-GFP, 35S::TaWRKY33-GFP and 35S::GFP control vectors were transiently expressed in wheat protoplasts. Scale bars = 10 μm

To further investigate their biological activities *TaWRKY1* and *TaWRKY33* were fused to the N-terminus of the green fluorescent protein (GFP) reporter gene under control of the CaMV 35S promoter and transferred into wheat mesophyll protoplasts. The 35S::GFP vector was transformed as the control. Fluorescence of TaWRKY1-GFP and TaWRKY33-GFP were specifically detected in the nucleus, whereas fluorescence of the control GFP was distributed throughout the cells (Fig. 4). Therefore, TaWRKY1 and TaWRKY33 likely function in the nucleus.

### Stress-related regulatory elements in the *TaWRKY1* and *TaWRKY33* promoters

To gain further insight into the mechanism of transcriptional regulation we isolated 2.0 kb promoter regions upstream of the *TaWRKY1* and *TaWRKY33* ATG start codons. We searched for putative *cis*-acting elements in the promoter regions using the databases Plant *Cis*-acting Elements, and PLACE (http://www.dna.affrc.go.jp/PLACE/) (Tables 2 and 3). A number of regulatory elements responding to drought, salt, low-temperature and ABA were recognized in both promoters, including ABA-responsive elements (ABREs), dehydration-responsive elements (DREs), W-box elements, and MYB and MYC binding sequences. In addition, gibberellin responsive elements (GAREs) and several elicitor responsive elements (ELREs) were identified (Tables 2 and 3).

**Table 2:** Putative *cis*-acting elements in the *TaWRKY1* and *TaWRKY33* promoters

Gene	ABRE	CBFHV	CCAAT-Box	DRE	DRE/CRT	DPBF	ELRE	GARE	LTRE	MYB	MYC	PYR	W-box	WRKY
Element														
*TaWRKY1*	17	2	2	0	1	3	2	4	2	26	26	0	9	9
*TaWRKY33*	9	5	3	2	2	3	4	2	4	24	8	3	23	16

**Table 3:** Functions of elements in the *TaWRKY1* and *TaWRKY33* promoters

Elements	Core sequence	Function
ABRE	ACGTG/ACGTSSSC/MACGYGB	ABA- and drought-responsive elements
CBFHV	RYCGAC	Drought- and cold-responsive elements
CCAAT-Box	CCAAT	Heat-responsive element
DRE	ACCGAGA/ACCGAC	ABA- and drought-responsive elements
DRE/CRT	RCCGAC	Drought-, high salt- and cold-responsive elements
DPBF	ACACNNG	Dehydration-responsive element
ELRE	TTGACC	Elicitor-responsive element
GARE	TAACAAR	GA-responsive element
LTRE	CCGAAA/CCGAC	Low-temperature responsive element
MYB	WAACCA/YAACKG/CTAACCA/CNGTTR/AACGG/TAACAAA/TAACAAA/MACCWAMC/CCWACC/GGATA	ABA- and drought-responsive elements
MYC	CATGTG/CANNTG	ABA- and drought-responsive elements
PYR	TTTTTTCC/CCTTTT	GA- and ABA-responsive elements
W-Box	TTTGACY/TTGAC/CTGACY/TGACY	SA-responsive element
WRKY	TGAC	Wound-responsive element

### Response mechanisms of *TaWRKY1* and *TaWRKT33* under abiotic stress

**Fig. 5:**
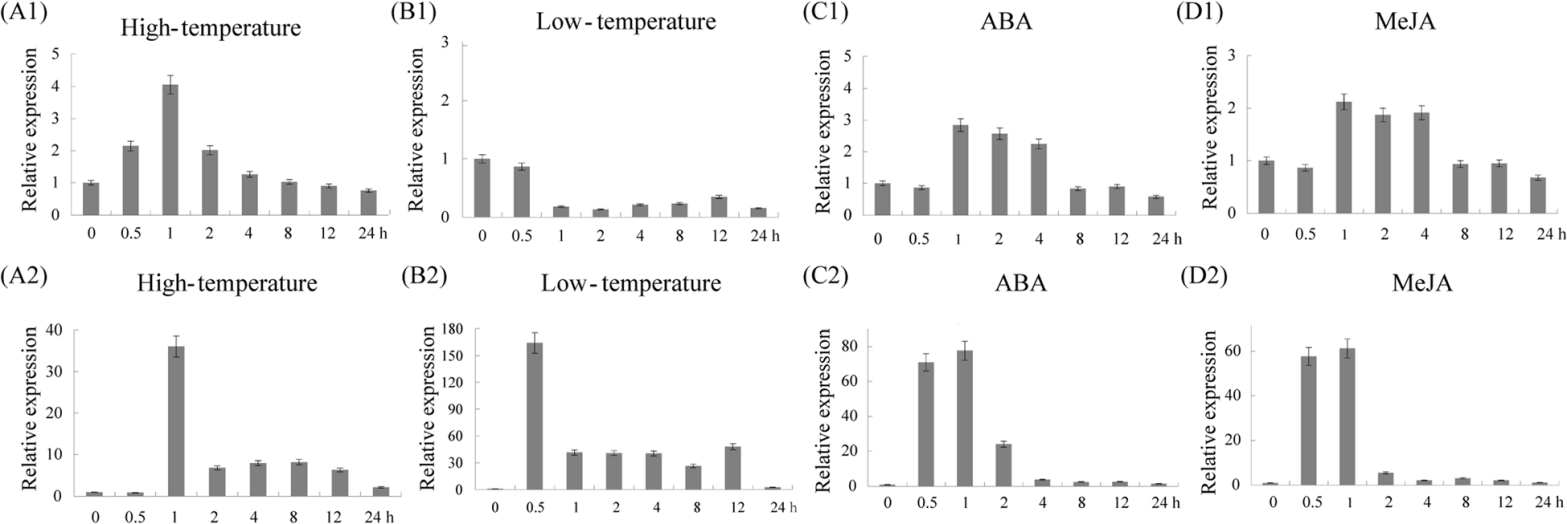
Expression patterns of *TaWRKY1* (**a1**–**d1**) and *TaWRKY33* (**a2**–**d2**) under abiotic stresses. The vertical ordinate is fold change; the horizontal ordinate is treatment time. The *actin* gene was used as an internal reference. The data are representative of three independent experiments

In order to clarify potential functions, the responses of *TaWRKY1* and *TaWRKY33* under various abiotic stress conditions were analyzed by qRT-PCR (Fig. 5). The *TaWRKY1* gene was slightly induced by high-temperature and exogenous ABA at a maximum level of about three-fold. Transcription of *TaWRKY1* was not affected by jasmonic acid methylester (MeJA), but was down-regulated by low-temperature.

By comparison, *TaWRKY33* rapidly responded to high-temperature, ABA and MeJA, with peak levels (more than 35-fold) occurring after one h of treatment. Low-temperature also activated transcription of *TaWRKY33*, with peak transcription levels earlier than those for drought, high-temperature, ABA and MeJA.

### Improved drought and ABA tolerance and decreased rates of water loss in transgenic *Arabidopsis*

**Fig. 6:**
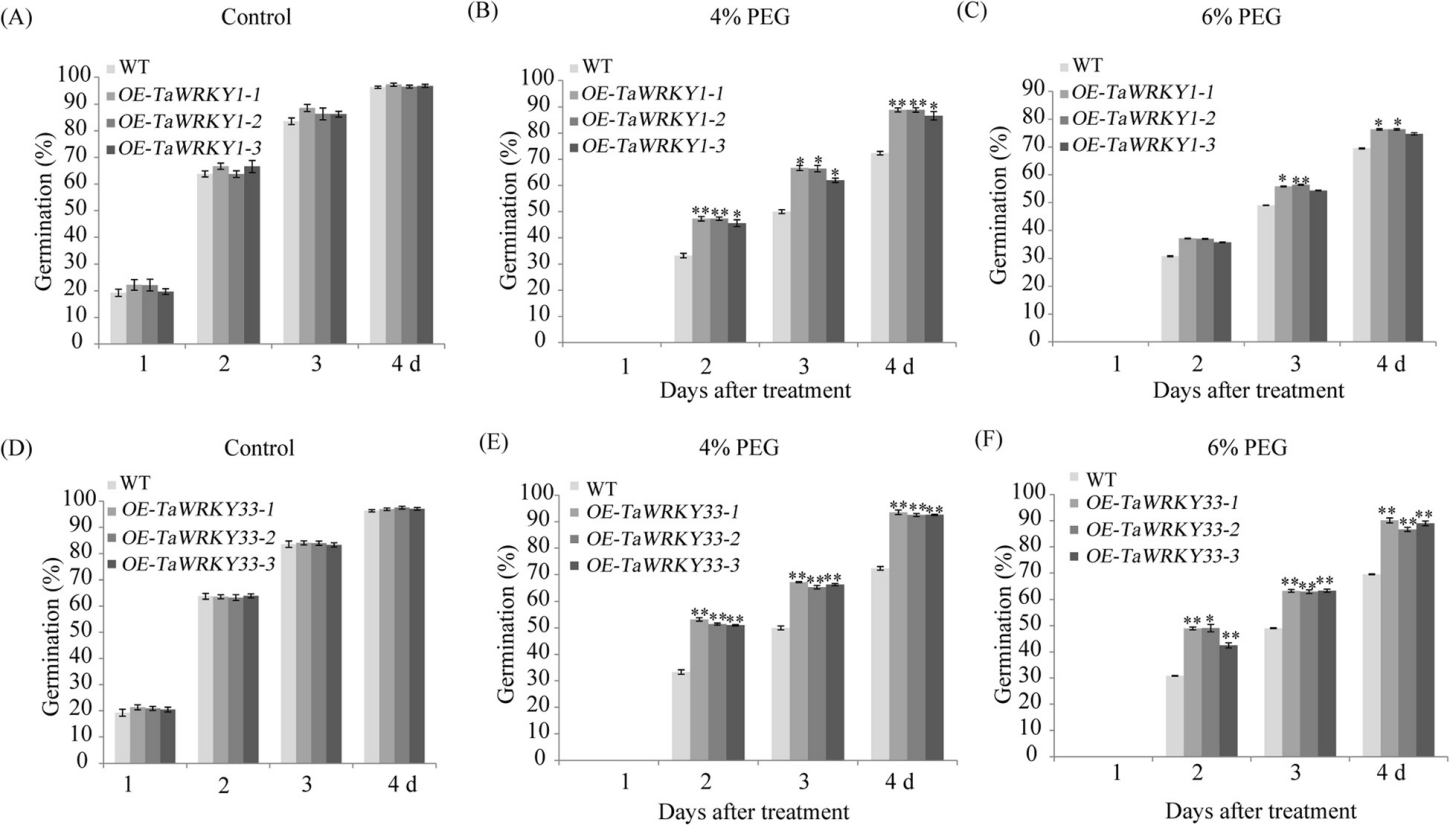
Germination of transgenic *Arabidopsis* lines under mock drought stress. Seed germinations of WT and *TaWRKY1* transgenic *Arabidopsis* lines on MS medium with or without 4 and 6 % PEG6000 (**a**-**c**). Seed germinations of WT and *TaWRKY33* transgenic *Arabidopsis* lines on MS medium with or without 4 and 6 % PEG6000 (**d**-**f**). Seeds were incubated at 4 °C for three days followed by 22 °C for germination. Seeds from three independent transgenic lines with *TaWRKY1* and *TaWRKY33* were grown on MS medium with or without 4 and 6 % PEG6000. WT seeds were grown in the same conditions as a control. Data are means ± SD of three independent experiments and * above the error bars or different letters above the columns indicate significant differences at *p* <0.05

WRKY transcription factors might be involved in plant stress signaling [51–53]. *TaWRKY1* and *TaWRKY33* under the control of CaMV35S were transformed into *Arabidopsis* plants to further investigate their functions. Semi-quantitative RT-PCR was used to confirm transgenic *Arabidopsis* plants carrying *TaWRKY1* and *TaWRKY33* genes (Additional file 2: Figure S1A). Progenies from transgenic lines were used for analysis of seed germination under osmotic stress. There was no difference in seed germination between transgenic lines and WT plants grown on Murashige and Skoog (MS) media (Fig. 6a and d). In comparison more than 88.7 % of *TaWRKY1* and *TaWRKY33* transgenic seeds germinated in 4 % polyethylene glycol 6000 (PEG6000)-supplemented MS media after four days compared to 72.4 % for WT seeds (Fig. 6b and e). In 6 % PEG6000-supplemented MS media (Fig. 6c) *TaWRKY1* transgenic seeds showed clear differences in germination rates compared to WT; nevertheless, *TaWRKY33* transgenic lines had higher germination rates than *TaWRKY1* transgenic lines and WT (Fig. 6f).

ABA tolerance of *TaWRKY1* and *TaWRKY33* transgenic lines was identified by seed germination rates of *Arabidopsis* on MS media containing ABA. Average germination rates of *TaWRKY1* transgenic lines were about 82 % compared to 75 % for WT in 0.5 μM ABA-supplemented MS media, meanwhile the germination rates of *TaWRKY33* transgenic lines were higher than those of the *TaWRKY1* transgenic lines and WT (Additional file 2: Figure S1C and S1F). Treated with 1 μM ABA, *TaWRKY33* transgenic lines exhibited obviously higher seed germination rates than those of WT, and *TaWRKY1* transgenic lines shared almost the same germination rates with WT (Additional file 2: Figure S1D and S1G).

**Fig. 7:**
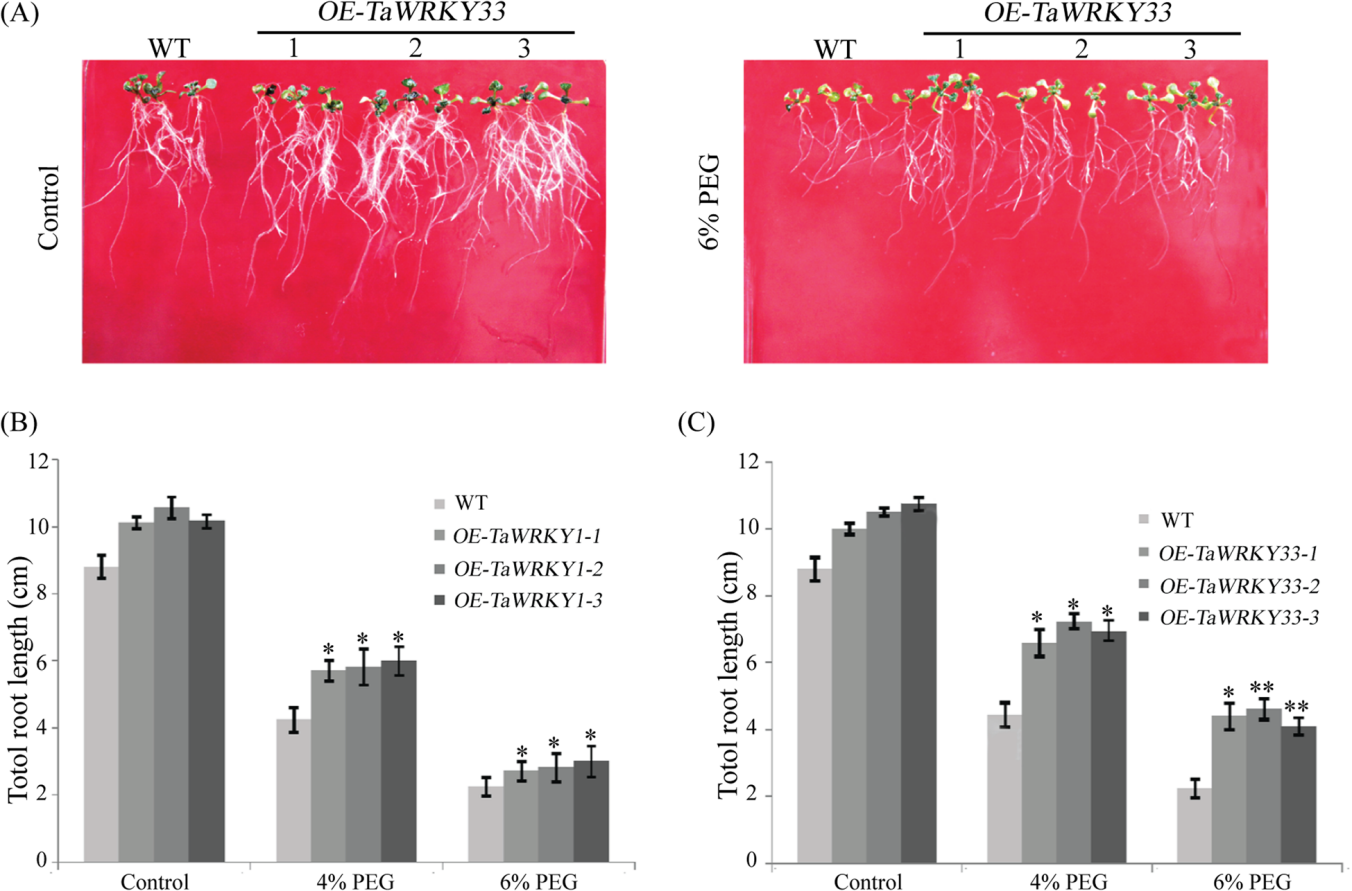
Total root lengths of transgenic *Arabidopsis* lines under mock drought stress. Phenotypes of WT and *TaWRKY33* transgenic *Arabidopsis* seedlings under MS medium with or without 6 % PEG6000 (**a**). Root lengths of WT and *TaWRKY1* transgenic *Arabidopsis* seedlings grown on MS medium with or without 4 and 6 % PEG6000 (**b**).  Root lengths of WT and *TaWRKY33* transgenic *Arabidopsis* seedlings grown on MS medium with or without 4 and 6 % PEG6000 (**c**). Five-day-old *Arabidopsis* seedlings were planted on MS medium with or without 4 and 6 % PEG6000 for seven days. Data are means ± SD of three independent experiments and * above the error bars or different letters above the columns indicate significant differences at *p* <0.05

Transgenic lines and WT *Arabidopsis* seeds were grown on MS medium for 5 days at 22 °C, and then transferred to MS medium containing 4 and 6 % PEG6000, respectively (Fig. 7 and Additional file 3: Figure S2). The *TaWRKY1* and *TaWRKY33* transgenic lines had similar phenotypes to WT seedlings under normal conditions. Total root lengths of the transgenic lines were longer than those of WT plants under both PEG6000 treatments after seven days, although PEG6000 stress reduced the growth of both transgenic and WT plants. *TaWRKY33* significantly promoted root growth in transgenic lines compared with *TaWRKY1* transgenic lines under PEG6000 treatment.

**Fig. 8:**
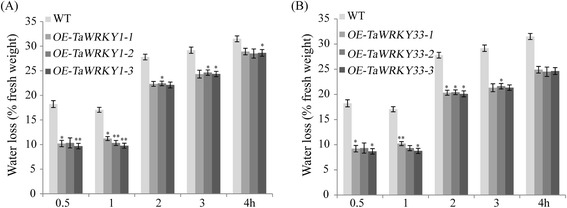
Determination of water loss of excised leaves from four-week-old *Arabidopsis*. The water loss of excised leaves of WT and *TaWRKY1* transgenic *Arabidopsis* lines (**a**). The water loss of excised leaves of WT and *TaWRKY33* transgenic *Arabidopsis* lines (**b**). Leaves at a similar stage from each line were used for the experiments. Data are means ± SD of three independent experiments and * above the error bars or different letters above the columns indicate significant differences at *p* <0.05

The transgenic lines showed lower rates of water loss compared with WT plants during dehydration treatment (Fig. 8). For example, rates of water loss of the *TaWRKY33* transgenics were less than 20.3 %, but *TaWRKY1* transgenic lines and WT plants lost 22.1 and 27.8 % after two h of dehydration, respectively (Fig. 8). These results showed that *TaWRKY33* transgenic lines had stronger water retaining capacity than WT plants.

### Enhanced thermotolerance of *TaWRKY33* transgenic lines

**Fig. 9:**
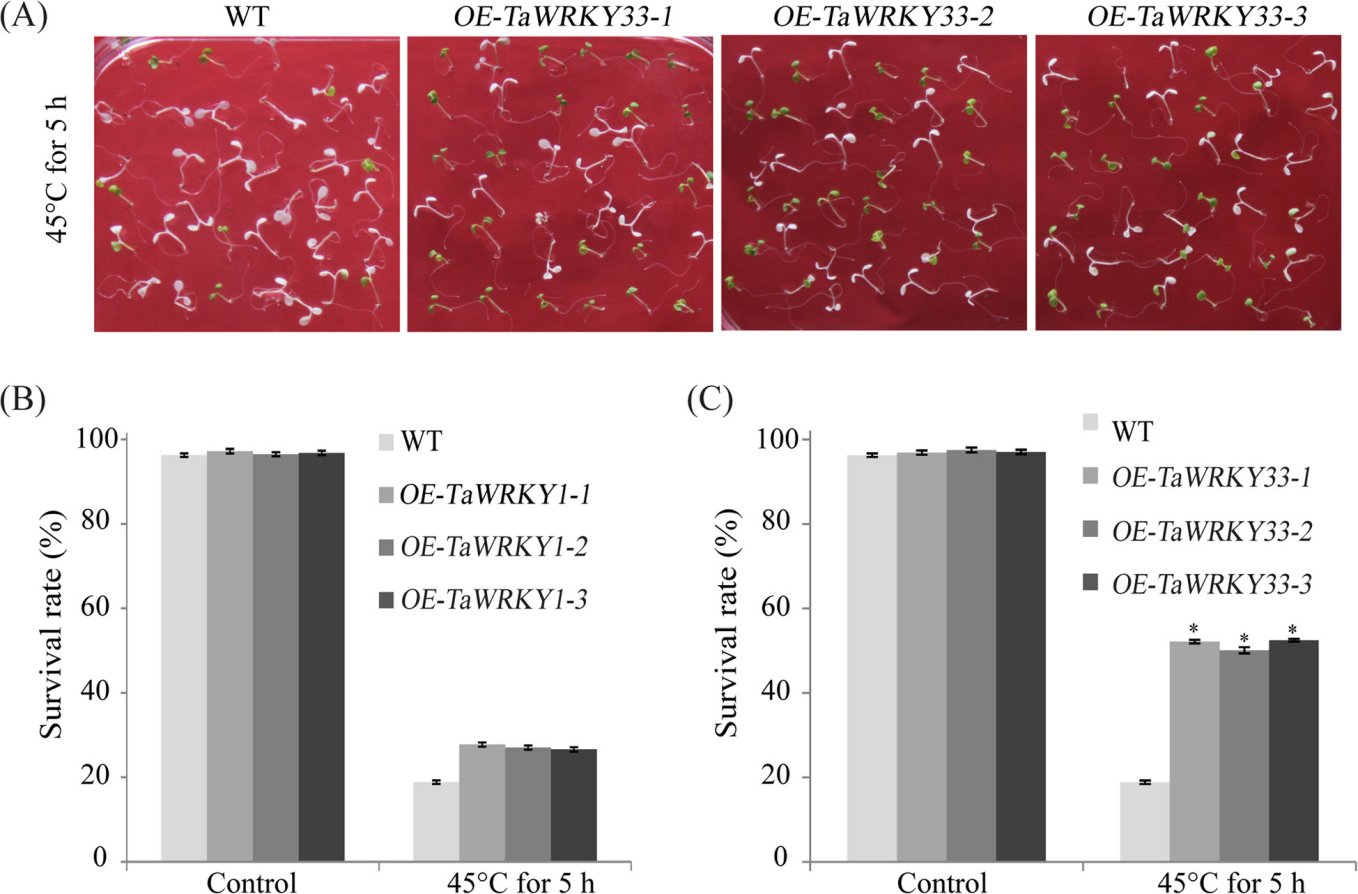
Analysis of transgenic *Arabidopsis* lines under heat stress. Phenotypes of WT and *TaWRKY33* transgenic *Arabidopsis* lines under heat stress (**a**). Sruvival rates of WT and *TaWRKY1* transgenic *Arabidopsis* lines under heat stress (**b**).  Sruvival rates of WT and *TaWRKY33* transgenic *Arabidopsis* lines under heat stress (**c**). Seeds from three independent transgenic lines of *TaWRKY1* and *TaWRKY33* were grown on MS medium. Five-day-old seedlings were heat-treated at 45 °C for five h before returning them to 22 °C to continue to grow vertically for 2 days. Data are means ± SD of three independent experiments and * above the error bars or different letters above the columns indicate significant differences at *p* <0.05

Following earlier results on response to high-temperature (Fig. 5) the functions of transgenic lines under high-temperature stress were investigated (Fig. 9). *TaWRKY33* transgenic lines exhibited high survival rates after exposure to 45 °C for five h, whereas *TaWRKY1* transgenic lines showed no clear differences from WT (Fig. 9). The survival rates of the *TaWRKY33* transgenic lines were more than 50 % after heat treatment compared to less than 30 % for *TaWRKY1* transgenics and WT. This suggested that *TaWRKY33* had a positive role in thermotolerance.

### Changed transcripts of stress-responsive genes

**Fig. 10:**
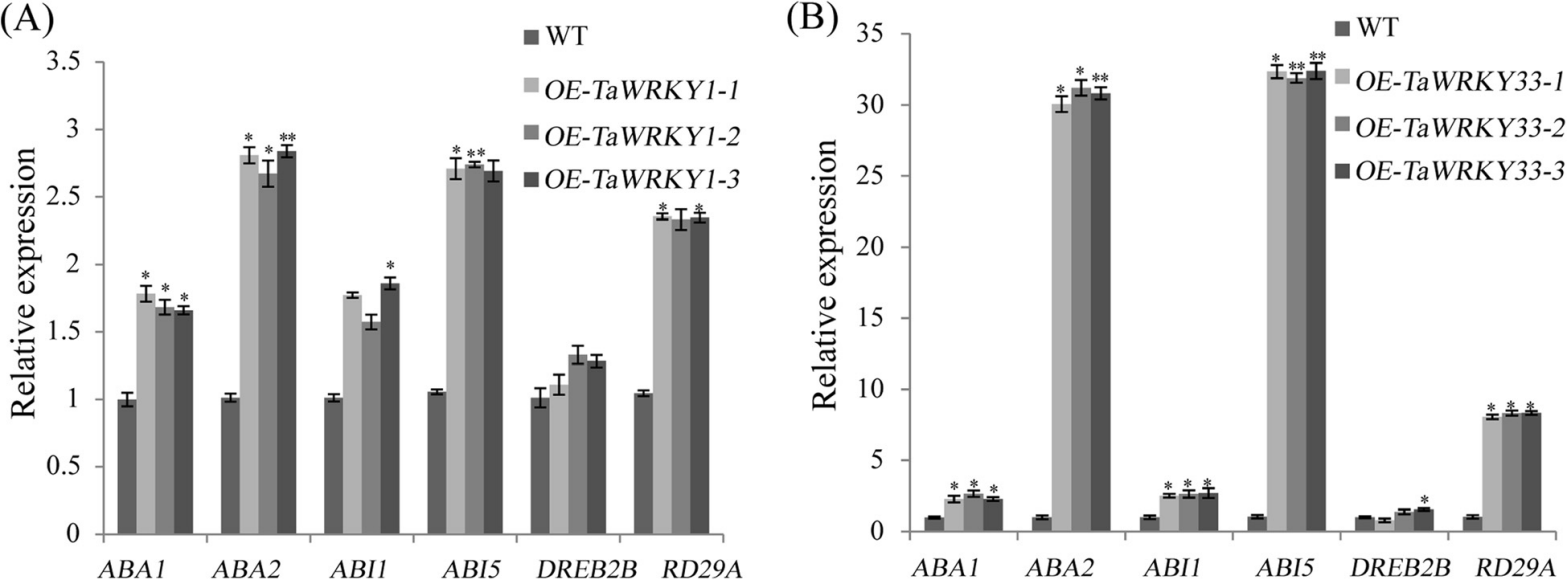
Expression levels of stress-responsive genes under regulation of *TaWRKY1* (**a**) and *TaWRKY33* (**b**). The vertical ordinates are fold changes, and the horizontal coordinates are gene names. Data are means ± SD of three independent experiments and * above the error bars or different letters above the columns indicate significant differences at *p* <0.05

**Fig. 11:**
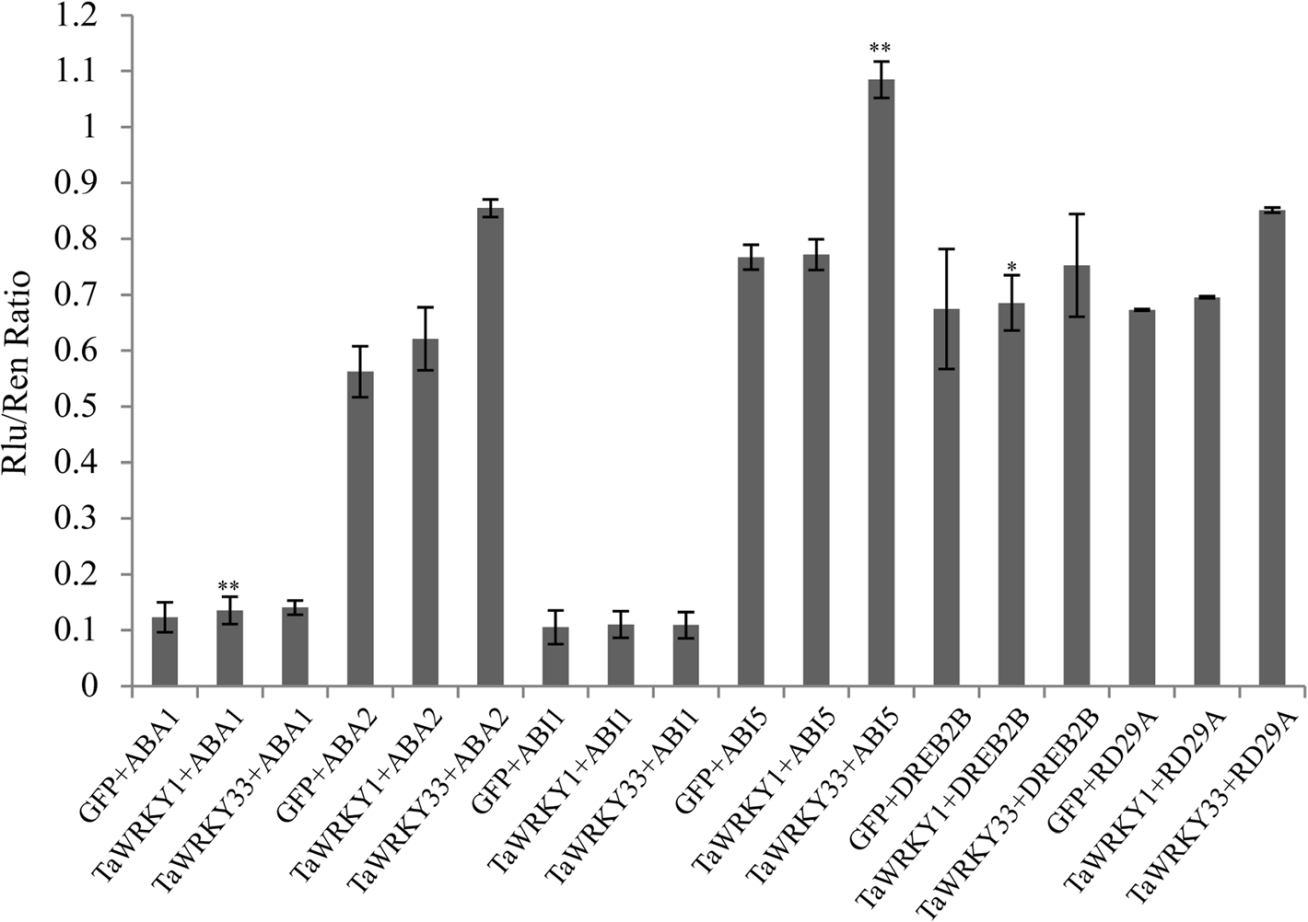
The activation of *Arabidopsis* promoters in transient Luciferase Assay. Co-operative activation of *ABA1*, *ABA2*, *ABI1*, *ABI5*, *RD29A* and *DREB2B* promoters from affected by *TaWRKY1* and *TaWRKY33* in a dual luciferase transient *Arabidopsis* transformation assays. Data are means ± SD of three independent experiments and * above the error bars or different letters above the columns indicate significant differences at *p* <0.05

*TaWRKY1* and *TaWRKY33* conferred stress tolerance in *Arabidopsis*. To investigate the tolerance mechanism we analyzed several stress-related genes possibly activated by *TaWRKY1* and *TaWRKY33*. Compared to WT, transcripts of *ABA1*, *ABA2*, *ABI1*, *ABI5* and *RD29A* were increased in *TaWRKY1* transgenics whereas *DREB2B* expression was not significantly changed under normal conditions (Fig. 10a). Similarly, overexpression of *TaWRKY33* regulated transcripts of *ABA1*, *ABA2*, *ABI1*, *ABI5*, *DREB2B* and *RD29A*, especially *ABA2* and *ABI5* to extremely high levels (Fig. 10b). As shown in Fig. 11, the LUC/REN ratio was increased significantly when the *ABA2* and *ABI5* pro-LUC reporter constructs were co-transfected with *TaWRKY33*, compared with the control that was co-transfected with the empty construct. These results indicated that overexpression of the *TaWRKY1* and *TaWRKY33* genes activated stress-responsive downstream genes.

## Discussion

The functions of WRKYs have been extensively explored in various plant species over the past ten years, especially in *Arabidopsis* and rice. Little information existed about the role of wheat WRKYs in mediating abiotic responses. Recently, Sezer et al. characterized 160 TaWRKYs according to sequence similarity, motif type and phylogenetic relationships, improving knowledge of WRKYs in wheat [54]. In the present study, 48 putative drought-responsive WRKY genes were identified from *de novo* transcriptome sequencing data of drought-treated wheat. The phylogenic tree revealed that most drought-responsive WRKYs belonged to Groups II and III (Fig. 1). Recent investigations showed that most WRKYs in these groups function in drought tolerance in many plant species. For example, *WRKY63*/*ABO3*, belonging to Group III, mediated responses to ABA and drought tolerance in *Arabidopsis* [55]. Similarly, *AtWRKY57* and *GmWRKY54*, which were identified as group II, were induced by drought and their expression conferred drought tolerance in *Arabidopsis* [48, 56]. In the present study *TaWRKY1* and *TaWRKY33*, members of Groups II and III, conferred drought tolerance in *Arabidopsis* (Figs. 6, 7, 8 and 9). Therefore, it was supposed that WRKYs in these groups might be involved in drought stress response.

WRKYs are important in many aspects of plant defense, including MAMP- (MTI) or PAMP-triggered (PTI) immunity, effector-triggered immunity (ETI) and systemin acquired resistance [56–64]. Increasing evidence shows that WRKYs are activated not only by disease-related stimuli and pathogen infection, but also by multiple abiotic stresses [17, 18, 52]. For example, 10 of 13 rice and 8 of 15 wheat WRKY genes responded to PEG6000, salt, cold or heat stresses [65, 66]. *TaWRKY44* may act as a positive regulator in drought, salt and osmotic stress responses [67]. Overexpression of *GhWRKY25* conferred tolerance to salt stress in tobacco [68]. In the present study, except for drought response, *TaWRKY33* was involved in strong responses to high- and low-temperature and ABA, possibly related to *cis*-elements in the promoter (Tables 2 and 3). For instance, the *TaWRKY33* promoter contained multiple ABRE and LTRE elements that might be responsible for low-temperature and exogenous ABA. The ELRE might induce large responses of *TaWRKY33* to abiotic stresses. In addition, *TaWRKY33* was highly induced by MeJA although there is no MeJA-related element. This could be the reason why MeJA-related elements had not been identified previously.

ABA is regarded to play a crucial role in plant abiotic stress response and development and is considered to be a negative regulator of biotrophic pathogen resistance [27, 69]. It has been reported that ABA-dependent and ABA-independent pathways exist in stress response [67]. *DREB2s* play important roles in ABA-independent pathway and often as marker genes in stress responses [70]. A number of transcription factors and their target genes are involved in mediating ABA signal transduction and have been shown to regulate many molecular and cellular responses [71]. Previous studies show that *ABI1*/*2* and *AtWRKY40* are key regulatory components of ABA receptors RCARs and ABAR, respectively. *ABI5*, a positive regulator of ABA signaling, exists in the downstream of *ABI1*/*2* and *AtWRKY40*. They are key players in ABA signal transduction and act by negatively regulating ABA response. ABA synthesis genes *ABA1* and *ABA2* were both detected in these studies, implying an acceleration of ABA production. Consistent with that, transcript abundance of *ABI5* also increased (Fig. 10), demonstrating that *TaWRKY33* likely increased the level of drought tolerance by increasing traffic through the ABA synthesis and transduction pathways.

It was reported that *RD29A* was induced by dehydration, low-temperature, high salinity or exogenous ABA. The promoter region of *RD29A* contains the *cis*-acting DRE that is involved in expression of *RD29A* rapidly responding to dehydration and high salinity stresses in *Arabidopsis*. Here, *RD29A* was up-regulated in *TaWRKY33* transgenic lines (Fig. 10), suggesting that *TaWRKY33* acts as a positive regulator in hyperosmotic stress response in *Arabidopsis*. These studies collectively demonstrated that *TaWRKY33* might play a key role in ABA- and drought-responsive signaling networks. Overexpression of wheat *TaWRKY2* enhanced *STZ* expression, whereas *TaWRKY19* promoted *DREB2A*-mediated activation of *RD29A*, *RD29B* and *Cor6.6*, resulting in tolerance to salt and drought in transgenic plants [49]. Therefore, wheat WRKYs affected stress tolerance through regulation of different downstream genes. Taken together, a model was proposed in which *TaWRKY1* and *TaWRKY33* transcription was activated under abiotic stress (Additional file 4: Figure S3). The MeJA-mediated signaling pathway is relevant to resistance to necrotrophic pathogens, wounding and insect herbivores [72–74]. The ABA and the JA could jointly modulate stress-related gene expression despite antagonistic interactions between the ABA and the JA/ET signaling pathways, they also [75]. In the present study, *TaWRKY33* was moderately and highly responsive to ABA and MeJA, respectively (Fig. 5). These results indicated that *TaWRKY33* could coordinately integrate the ABA and the MeJA pathways, but not antagonize them. Therefore, we speculate that *TaWRKY33* might have roles in interaction of the ABA and MeJA signaling pathways and might be related to both abiotic stress tolerance and disease responses in plants.

## Conclusions

Forty-eight putative drought-responsive WRKY genes were identified from *de novo* transcriptome sequencing data of drought-treated wheat. They were classified into three groups, according to sequence similarity and motif identity. *TaWRKY1* and *TaWRKY33*, belonging to Groups II and III, were selected for further investigation. Both *TaWRKY1* and *TaWRKY33* responded to multiple stresses. Overexpression of *TaWRKY1* and *TaWRKY33* activated several stress-related downstream genes, increased germination rates and promoted root growth in *Arabidopsis* under stresses. These studies provide candidate genes for future functional analysis of TaWRKYs involved in the drought- and heat-related signal pathways in wheat.

## Methods

### *De novo* sequencing of drought-treated wheat

Total RNA was isolated using TRIzol reagent (Invitrogen) and treated with RNase free DNase I (Qiagen). Poly (A) mRNA was purified from total RNA using oligo (dT) magnetic beads and fragmented into small pieces using divalent cations. First-strand cDNA was generated using reverse transcriptase and random primers. This was followed by synthesis of the second-strand cDNA. Then, single-end and paired-end RNA-seq libraries were prepared following Illumina’s protocols and sequenced on the Illumina GA II platform [76, 77].

*De novo* assembly of the short reads was performed using SOAPdenovo software (http://soap.genomics.org.cn), which adopts the de Bruijn graph data structure that is sensitive to the sequencing error to construct contigs [78]. According to the overlap information in the short reads, the reads were then realigned to the contig sequence with high coverage, and the paired-end relationship between reads was transferred to linkage between contigs. Unreliable linkages between two contigs were filtered and the remaining contigs with compatible connections to each other, and having at least three read-pairs, were constructed into scaffolds. We constructed scaffolds starting with short paired-ends and then iterated the scaffolding process, step by step, using longer insert size paired-ends. To fill the intra-scaffold gaps, we used paired-end information to retrieve read pairs that had one read with one end mapped to the contigs and another read located in the gap region, and then did a local assembly with the unmapped end to extend the contig sequence in the small gaps in the scaffolds.

Gene expression profiling was measured by mapping reads to assembled sequences using SOAP [79]. The most widely used approach is to count uniquely mapped reads. Then the FPKM value for each transcript was measured in Fragments Per kb per Million fragments [80]. We then used the False Discovery Rate (FDR) method to determine the threshold of the *p*-value in multiple tests. FDR ≤ 0.001 and a relative change threshold of two-fold were used to judge the significance of differentiated gene expression. The analysis firstly maps all differentially expressed genes (DEGs) to GO terms in the database by virtue of calculating gene numbers for every term, followed by an ultra-geometric test to find significantly enriched GO terms in DEGs compared to the transcriptome background. The calculated p- value was subjected to a Bonferroni Correction, taking a corrected *p*-value of 0.05 as a threshold. GO terms fulfilling this condition were defined as significantly enriched GO terms in DEGs [81]. For pathway enrichment analysis, we mapped all DEGs to terms in KEGG database.

### Plant materials and stress treatments

Wheat (*T. aestivum cv.* Xiaobaimai) seedlings which were provided by Dr Rui-Lian Jing (Institute of Crop Science, Chinese Academy of Agricultural Sciences) were grown in Hoagland’s liquid medium at 22 °C under a 16 h light/8 h darkness photoperiod. Ten-day-old seedlings were used for dehydration, high-temperature, low-temperature, MeJA and ABA treatments. Seedlings on filter paper were exposed to air for induction of rapid drought conditions, or placed in 4 and 42 °C chambers for low and high-temperature treatments, respectively. For dehydration treatment, seedlings were transferred to filter paper and dried at 25 °C under normal conditions. For MeJA and ABA treatments, seedling roots were immersed in solutions containing 100 μM MeJA and 100 μM ABA, respectively. The samples were harvested at 0, 0.5, 1, 2, 4, 8, 12 and 24 h.

### RNA extraction and qRT-PCR analyses

Total RNA was extracted using Trizol reagent according to the manufacturer’s protocol (TIANGEN, China) and treated with DNase I (TaKaRa, Japan) to remove genomic DNA contamination. First strand cDNA was synthesized using a PrimeScript First-Strand cDNA Synthesis Kit (TaKaRa) following the manufacturer’s instructions. qRT-PCR was conducted using an ABI Prism 7500 system (Applied Biosystems, Foster City, CA). The *actin* gene was used as an internal control for normalization of template cDNA. Each PCR was repeated three times in total volumes of 20 μl containing 2 × Taq PCR Master Mix (TIANGEN). Validation experiments were performed to demonstrate that amplification efficiencies of the *TaWRKY1-* and *TaWRKY33*-specific primers were approximately equal to the amplification efficiency of the endogenous reference primers. Quantitative and data analyes were performed as previously described [82].

### Gene isolation and sequence analysis

Open reading frames of *TaWRKY1* (Genbank No. KT285206) and *TaWRKY33* (Genbank No. KT285207) were amplified by PCR using specific primers. PCR products were cloned into pEASY-T1 vectors (TransGen, China) and sequenced with an ABI 3730XL 96-capillary DNA analyzer (Lifetech, America).

Maximum likelihood was used to construct phylogenetic trees by the MEGA5.1 program, and the confidence levels of monophyletic groups were estimated using bootstrap analyses of 1000 replicates [83].

Predicted protein domains of TaWRKY1 and TaWRKY33 were identified by the SMART tool (http://smart.embl-heidelberg.de/), and their tertiary structures were obtained using the Phyre2 tool (http://www.sbg.bio.ic.ac.uk/phyre2).

### Plasmid construction for subcellular localization analysis

The open reading frames of *TaWRKY1* and *TaWRKY33* were inserted into N-terminal GFP protein driven by the CaMV 35S promoter of subcellular localization vector p16318 [83]. For transient expression assays, mesophyll protoplasts were isolated, transfected with p16318::*TaWRKY1* and p16318::*TaWRKY33*, and GFP fluorescence signals were observed with a confocal laser scanning microscope (Nikon, Japan). FM4-64 dye (Molecular Probes, Carlsbad, CA) was excitated at 543 nm and fluorescence was recorded using a 650 nm long pass filter. All transient expression experiments were repeated three times [84].

### Transient luciferase assay in *Arabidopsis*

For the analysis of transcription activities of TaWRKY1 and TaWRKY33 in response to *ABA1*, *ABA2*, *ABI1*, *ABI5*, *RD29A* and *DREB2B* promoters, the 2.5 kb promoter regions were cloned into the transient expression reporter vector pGreenII 0800-LUC which contains the CaMV 35S promoter-REN cassette and the promoterless-LUC cassette, respectively [85, 86]. The *TaWRKY1* and *TaWRKY33* genes were cloned into N-terminal GFP protein driven by the CaMV 35S promoter. The constructed effectors and reporter plasmids were transfected into mesophyll protoplasts of *Arabidopsis* Columbia-0 which were collected by our own laboratory. Transfected protoplasts were incubated in darkness at 22 °C. Firefly luciferase and renilla luciferase were assayed using the dual luciferase assay reagents (Promega, USA). Data was collected as the ratio of LUC/REN. All transient expression experiments were repeated three times.

### Generation, and stress treatments of transgenic *Arabidopsis*

The coding sequences of *TaWRKY1* and *TaWRKY33* were cloned into pBI121 under control of the CaMV 35S promoter, resulting in 35S::*TaWRKY1* and 35S::*TaWRKY33* constructs. These constructs were confirmed by sequencing and then separately used in transformation mediated by *Agrobacterium* (*Agrobacterium tumefaciens*) to obtain three transgenic *Arabidopsis* lines. Kanamycin-resistant *Arabidopsis* transformants carrying *TaWRKY1* and *TaWRKY33* were generated using the vacuum infiltration method [86]. Transformed plants were cultured on MS medium containing 0.8 % agar and 50 mM Kanamycin in a day/night regime of 16/8 h under white light (with 50 photons m-1 s-1) at 22 °C for 2 weeks and then transferred to soil.

Homozygous T3 seeds of transgenic lines were used for phenotypic analysis. *Arabidopsis* seeds were grown on 10 × 10 cm MS agar plates that were routinely kept for three days in darkness at 4 °C to break dormancy and transferred to a tissue culture room under a day/night regime of 16/8 h under white light (with 50 photons m-1 s-1) at 22 °C for five days. For the germination assay, seeds were subjected to 4 or 6 % (w/v) PEG6000, and 0.5 or 1 μM ABA treatments. For drought treatment, 5-day-old seedlings were transferred to MS agar plates containing 4 and 6 % PEG6000 for seven days. Total root lengths of the *Arabidopsis* plants were measured [87]. Five-day-old seedlings were heat-treated at 45 °C for five h before returning them to 22 °C to continue to grow vertically for two days. Seeds were considered germinated when radicles had completely emerged from the seed coat. All measurements were repeated three times.

## Abbreviations

ABA, abscisic acid; ABRE, ABA-responsive element; DEGs, differentially expressed genes; DRE, dehydration-responsive elements; ELRE, elicitor responsive element; FDR, false discovery rate; GFP, green fluorescent protein; MeJA, jasmonic acid methylester; PEG6000, polyethylene glycol 6000; qRT-PCR, quantitative real-time PCR; WT, wild type.

## Additional files

Additional file 1: Table S1. Nucleic acid sequences of drought-induced responsive WRKY genes in wheat. (XLSX 24 kb) Additional file 2: Figure S1. Detection of the expression levels of *TaWRKY1* and *TaWRKY33* transgenic *Arabidopsis* lines (A). Germination of transgenic *Arabidopsis* lines under ABA stress (B-G). (TIF 527 kb) Additional file 3: Figure S2. Phenotypes of *TaWRKY1* transgenics. (TIF 928 kb) Additional file 4: Figure S3. Probable modes of action of TaWRKY1 and TaWRKY33. (TIF 228 kb)
