# Supplementary figures and images for: Drought-responsive WRKY transcription factor genes TaWRKY1 and TaWRKY33 from wheat confer drought and/or heat resistance in Arabidopsis
Source: BMC Plant Biol. 2016 May 23;16:116. doi: 10.1186/s12870-016-0806-4 (PMC4877946; doi:10.1186/s12870-016-0806-4)

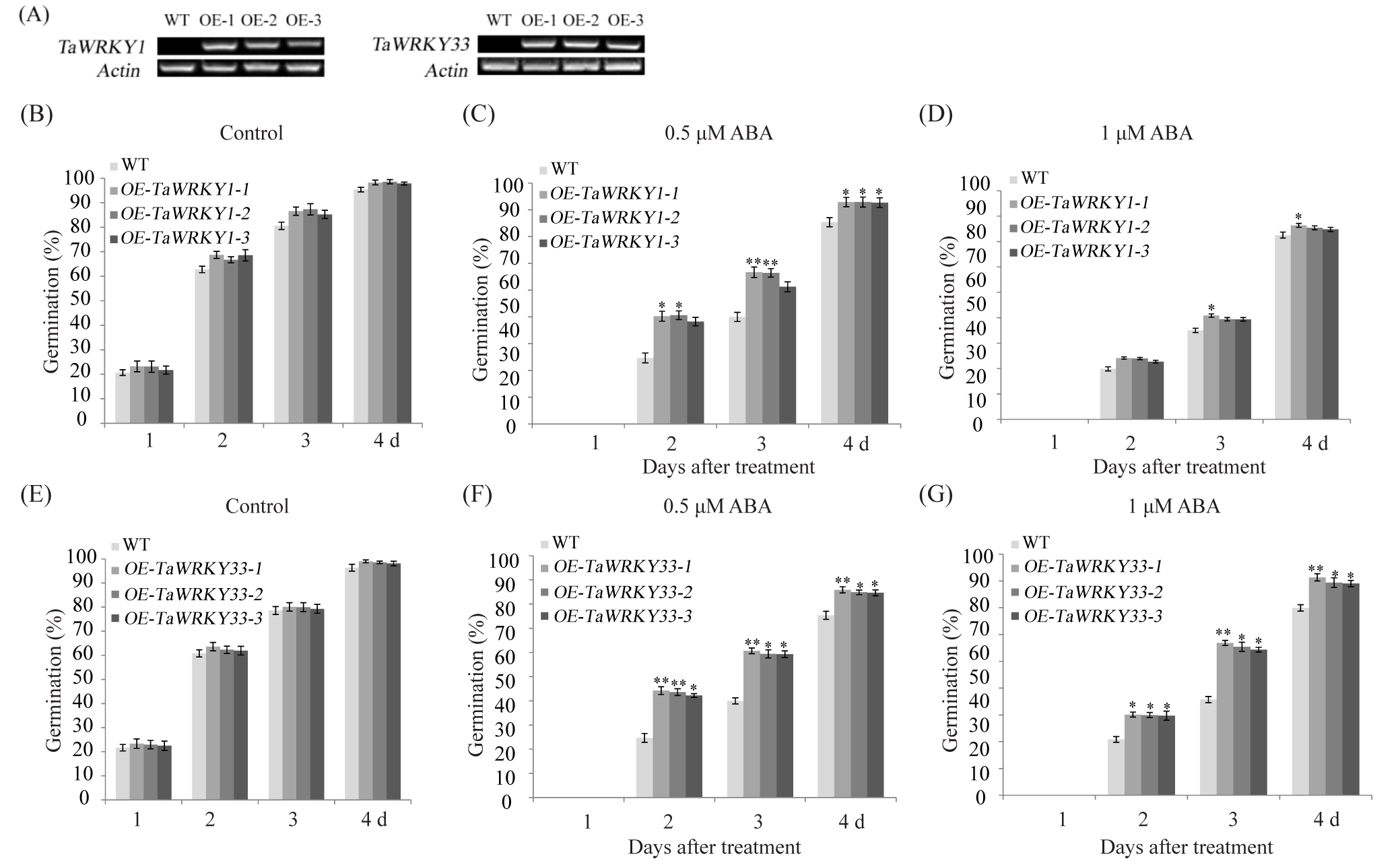

Supplement: Additional file 2: Figure S1. — Detection of the expression levels of TaWRKY1 and TaWRKY33 transgenic Arabidopsis lines (A). Germination of transgenic Arabidopsis lines under ABA stress (B-G). (TIF 527 kb) [file 12870_2016_806_MOESM2_ESM.tif]

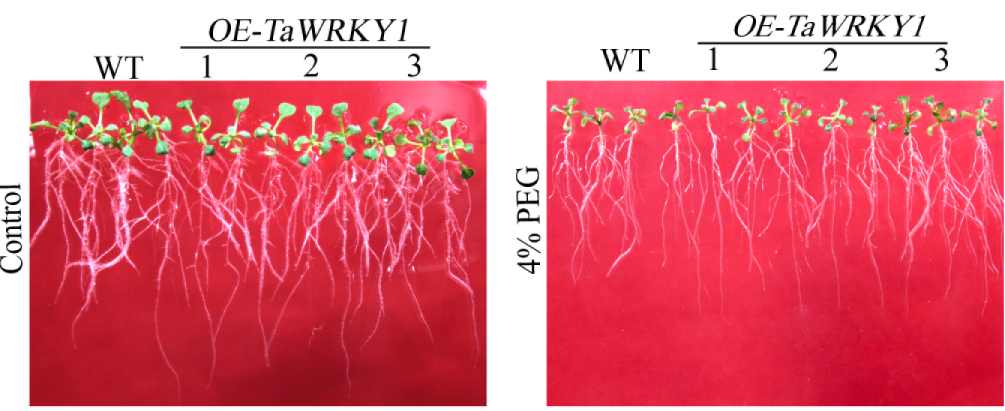

Supplement: Additional file 3: Figure S2. — Phenotypes of TaWRKY1 transgenics. (TIF 928 kb) [file 12870_2016_806_MOESM3_ESM.tif]

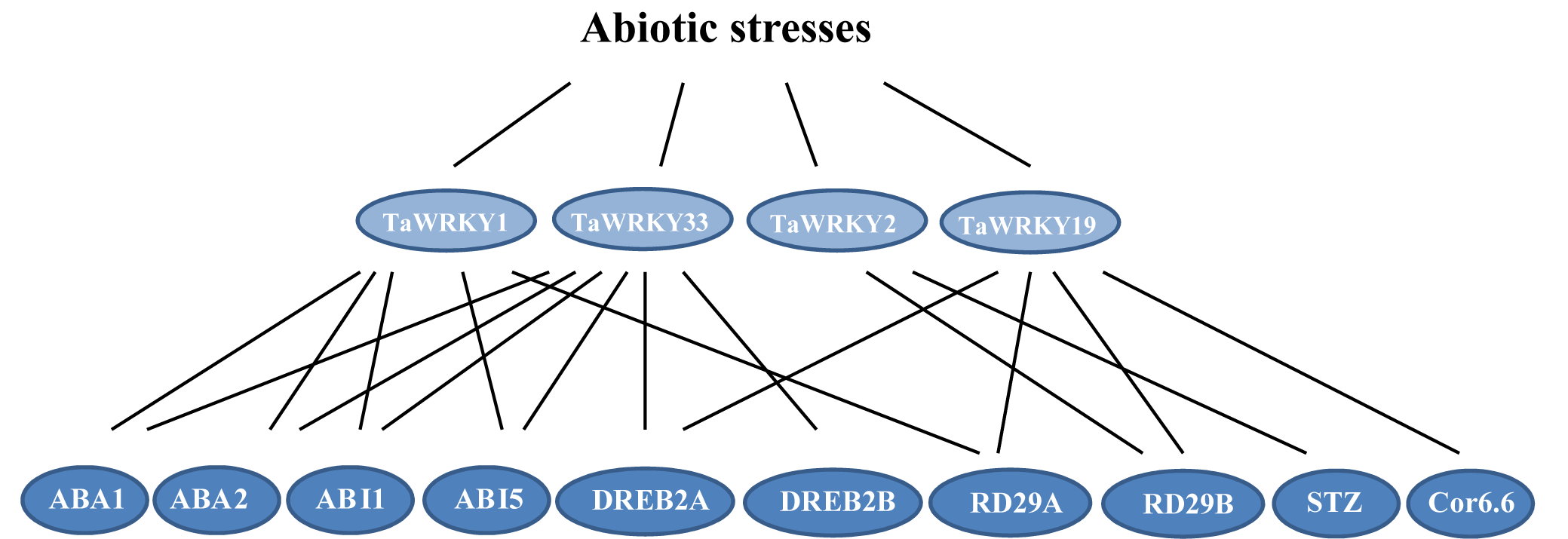

Supplement: Additional file 4: Figure S3. — Probable modes of action of TaWRKY1 and TaWRKY33. (TIF 228 kb) [file 12870_2016_806_MOESM4_ESM.tif]
